# Dynamics of Macronutrient Uptake and Removal by Modern Peanut Cultivars

**DOI:** 10.3390/plants10102167

**Published:** 2021-10-13

**Authors:** Carlos Alexandre Costa Crusciol, José Roberto Portugal, João William Bossolani, Luiz Gustavo Moretti, Adalton Mazetti Fernandes, Jader Luis Nantes Garcia, Gleize Leviski de Brito Garcia, Cristiane Pilon, Heitor Cantarella

**Affiliations:** 1 Department of Crop Science, College of Agricultural Sciences, São Paulo State University (UNESP), Botucatu 18610-034, SP, Brazil; jose.portugal@unesp.br (J.R.P.); bossolani.agro@gmail.com (J.W.B.); souzamoretti@gmail.com (L.G.M.); 2 Center of Tropical Roots and Starches (CERAT), São Paulo State University (UNESP), Botucatu 18610-034, SP, Brazil; adalton.fernandes@unesp.br; 3 Department of Forest, Soil and Environmental Sciences, College of Agricultural Sciences, São Paulo State University (UNESP), Botucatu 18610-034, SP, Brazil; jader_nantes@hotmail.com (J.L.N.G.); gleizeleviskidebritogarcia@gmail.com (G.L.d.B.G.); 4 College of Agricultural and Environmental Science, University of Georgia, 2356 Rainwater Road Tifton, Athens, GA 31793, USA; cpilon@uga.edu; 5 Soils and Environmental Resources Center, Agronomic Institute of Campinas (IAC), Av. Barão de Itapura 1481, Campinas 13020-902, SP, Brazil

**Keywords:** *Arachis hypogaea* L., plant growth, plant nutrition, mineral absorption

## Abstract

The productive potential of new peanut cultivars has increased over the years in relation to old cultivars, especially when compared with ones with upright growth habit. Thus, the requirement for macronutrients for these new cultivars may also have increased, making the existing fertilizer recommendation tables obsolete, thus increasing the need for further studies measuring the real macronutrient requirements of these new peanut cultivars. Our study aimed to evaluate the growth patterns and the macronutrient absorption rate throughout the biological cycle of three modern runner peanut cultivars, as well as the potential for producing dry matter, pods, and kernels, and their respective macronutrient accumulations. The experimental design was a randomized complete block with split-plots and nine replications. The experimental plots consisted of three peanut cultivars (IAC Runner 886, IAC 505, and IAC OL3), and subplots consisted of nine plant samplings (14, 28, 42, 56, 70, 84, 105, 126, and 147 days after emergence (DAE)). Our results showed that modern peanut cultivars presented nutrient accumulation around 30 to 40 days earlier than older cultivars, as well as increasing the uptake by K and Ca. IAC 505 absorbed higher amounts of macronutrients and resulted in greater dry matter production compared with IAC OL3 and IAC Runner 886. Our study demonstrated that the most appropriate time for plants to find greater availability of nutrients in the soil is 70 to 84 DAE, in addition to highlighting the need for updates on nutritional recommendations for higher yields of modern peanut cultivars.

## 1. Introduction

Peanuts (*Arachis hypogaea* L.) are one of the most important food legumes and oilseed crops worldwide, offering multiple benefits to human nutrition, especially as they have a nutrient composition similar to that of tree nuts, in addition to serving numerous industrial sectors [1,2]. From an economic perspective, peanuts are cultivated in more than 100 countries around the world, spread over tropical, sub-subtropical, and warm temperate climates [3], for a total of approximately 28 million ha [4]. Brazil occupies a prominent position in worldwide peanut production, especially grown in crop rotation with sugarcane to improve soil fertility for the renovation of sugarcane fields [5] or in degraded pasture areas to recover the productive potential of forage grown after peanuts [6,7]. However, in both situations, the proper use of peanut fertilization is necessary, as this practice directly influences the crop yield [8].

The peanut production system differs from other crops with regard to soil fertility, since it is a consensus among researchers and producers that this crop efficiently takes advantage of the residual effect of previous crops [7,9]. For this reason, peanut producers have been negligent in the soil fertilization program in fields grown with peanuts. However, determining the responses of peanuts to fertilization is important in the proper implementation of this practice [10]. For the proper management of fertilization, it is essential to apply the correct dose and correct time, among others [11]. In Brazil, for peanut cultivation normally nitrogen is supplied by biological fixation, phosphorus is supplied up to 100 kg ha^−1^ P_2_O_5_ and potassium up to 60 kg ha^−1^ of K_2_O, both at sowing, while calcium and magnesium are provided by liming and sulfur about 20 kg ha^−1^ of S [12]. However, any fertilization recommendation must be based on knowing the rate of nutrient accumulation by plants [13].

Information about nutrient uptake rates during the biological cycle of peanuts in Brazil was established through a series of research studies, mostly performed more than two decades ago [9] with old cultivars, which are no longer in the market and had low productive potential compared with new genetic materials. Among the four types of peanuts (Virginia, runner, Spanish, and Valencia), the runner cultivars are the main ones cultivated in the United States and Brazil, which are among the main producers in the world [14,15]. Recently, high oleic type cultivars have expanded their production area [16]. The development of more productive genotypes with higher levels of protein and oil increased the demand for more fertile soils so that the maximum potential of the crop is reached [5]. Plant nutritional balance combined with proper nutrient supply can maximize productivity and quality of peanuts, ensuring an adequate supply of this oilseed in the consumer market and reducing losses in the cropping system [17].

Therefore, information available in official fertilizer recommendation reports is out of date, underestimating the productive potential of the new cultivars. Updates in peanut fertilizer recommendation systems are highly necessary in view of the increase in the population and worldwide consumption of peanuts and their derivatives. Therefore, our study aimed to quantify the accumulation and distribution of dry matter, as well as the accumulation and translocation of macronutrients throughout the biological cycle of three modern cultivars (IAC Runner 886, IAC 505, and IAC OL3) considered as the most cultivated in Brazil.

## 2. Results

The contents of N, P, and S in the peanut leaves did not differ among cultivars (Table 1). K and Ca leaf contents were lowest in IAC OL3, whereas Mg content was highest compared with the other two cultivars.

**Figure 1:**
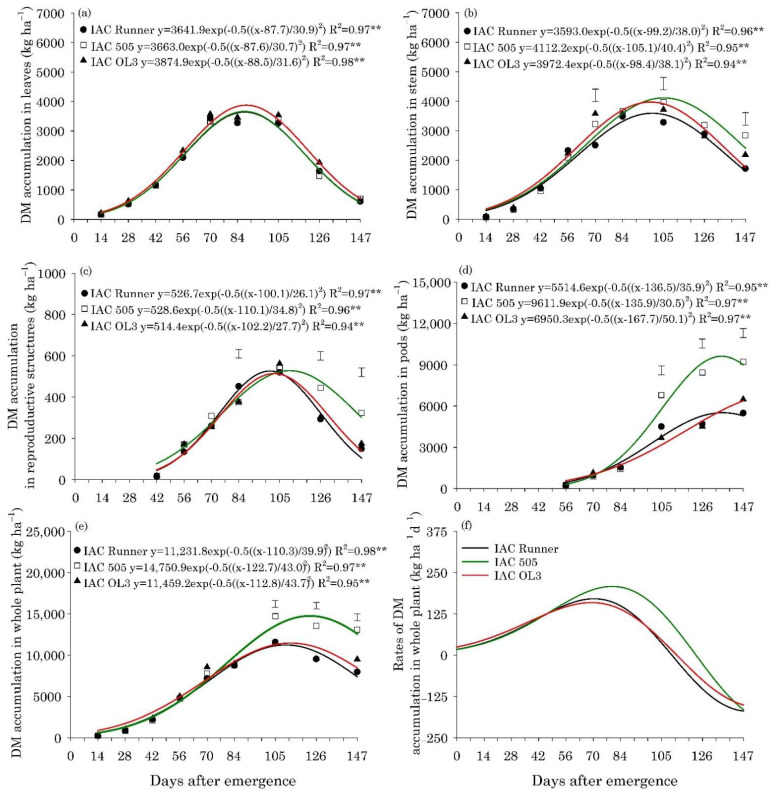
Dry matter (DM) accumulation in leaves (**a**), stems (**b**), reproductive structures (**c**), pods (**d**), and whole plant (**e**), and DM accumulation rates in the whole plant (**f**) of peanut cultivars throughout the season. ** is: significant at *p* ≤ 0.01, by the F test. Vertical bars indicate the least significant difference values according to the LSD test at *p* ≤ 0.05.

The peak in leaf DM accumulation occurred 88 days after emergence (DAE) for the three cultivars, with an average of approximately 3700 kg ha^−1^, and the means did not differ among the cultivars in any of the sampling dates evaluated (Figure 1a). For the stem DM accumulation, the period of greatest contribution occurred between 98 and 105 DAE for the three cultivars (Figure 1b). Differences among cultivars were observed after 70 DAE, indicating that the initial growth across the cultivars was similar and slow. From 105 DAE until the end of the season, cultivar IAC 505 was superior in mass accumulation compared with IAC Runner and similar to IAC OL3 (Figure 1b). The greatest DM accumulation in the reproductive structures occurred 100 DAE for the cultivars IAC Runner and IAC OL3, while it was 110 DAE for IAC 505 (Figure 1c). Pod DM accumulation increased until close to the end of the season, followed by a slight decrease in DM accumulated in the pods (Figure 1d).

Clearly, the amount of phytomass in the pods of IAC 505 was greater than that in the other cultivars, especially after 105 DAE. Additionally, at 147 DAE, pod DM accumulation was approximately 70% and 40% higher in IAC 505 than that in IAC Runner and IAC OL3, respectively. The total DM accumulation in peanut plants was similar among cultivars up to 84 DAE; however, from 105 DAE, cultivar IAC 505 had higher DM accumulation in the whole plant until the end of the season (Figure 1e).

The maximum DM accumulation in the plants of IAC 505 (14,750 kg ha^−1^) was greater than IAC Runner (11,232 kg ha^−1^) and IAC OL3 (11,459 kg ha^−1^), which occurred at 123, 110 and 113 DAE for IAC 505, IAC Runner and IAC OL3, respectively (Figure 1f). In general, the rate of DM accumulation in the whole peanut plant was highest for IAC 505 than the other cultivars, which had similar values. IAC 505 had values about 208 kg ha^−1^ d^−1^, whereas IAC Runner and IAC OL3 indicated a rate of maximum DM accumulation of 159 and 170 kg ha^−1^ d^−1^, respectively (Figure 1f).

**Figure 2:**
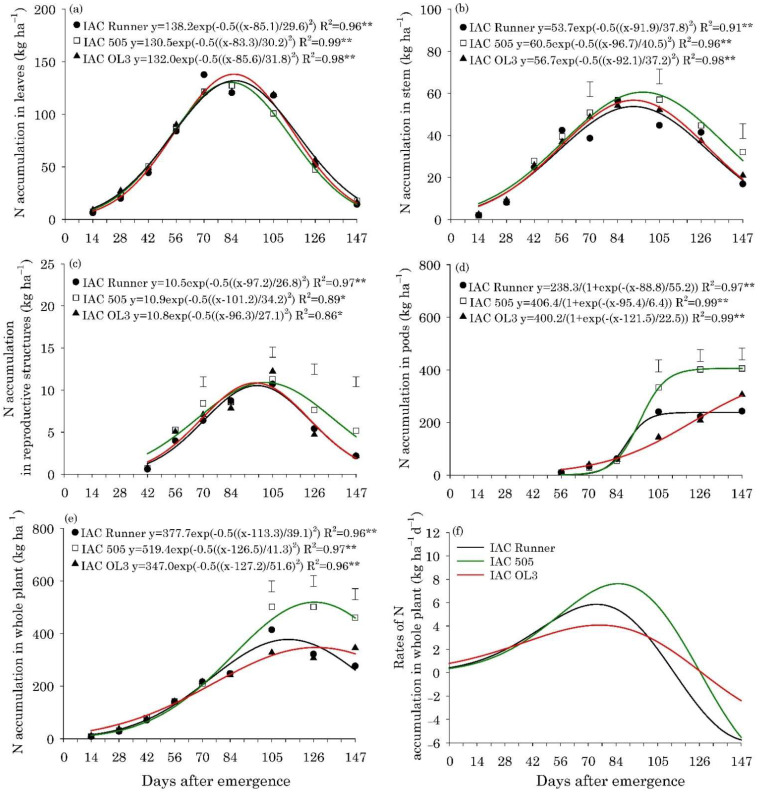
Nitrogen (N) accumulation in leaves (**a**), stems (**b**), reproductive structures (**c**), pods (**d**), and whole plant (**e**), and N accumulation rates in the whole plant (**f**) of peanut cultivars throughout the season. ** and * are: significant at *p* ≤ 0.01 and *p* ≤ 0.05, respectively, by the F test. Vertical bars indicate the least significant difference values according to the LSD test at *p* ≤ 0.05.

**Figure 3:**
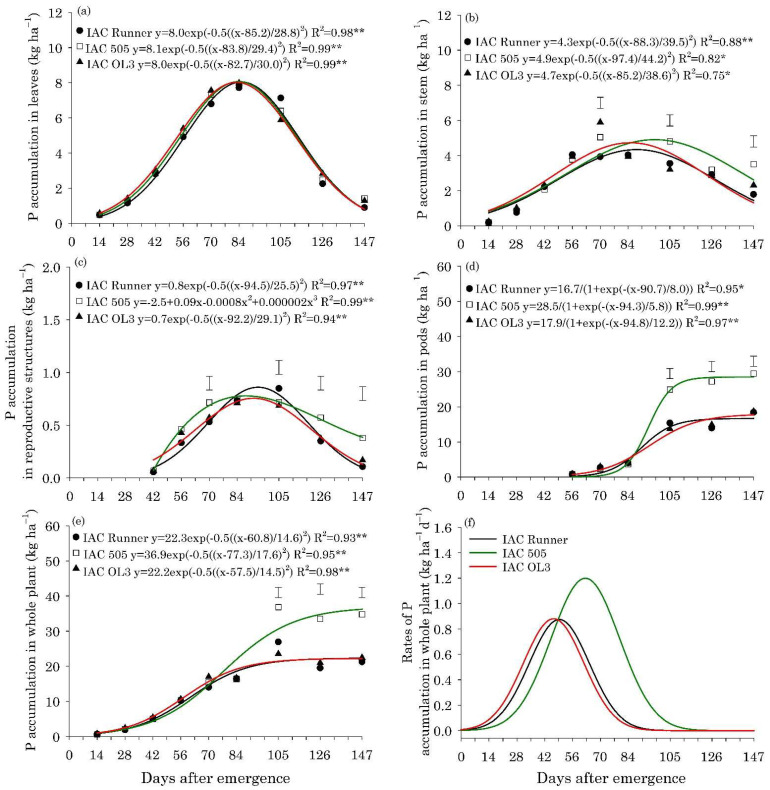
Phosphorus (P) accumulation in leaves (**a**), stems (**b**), reproductive structures (**c**), pods (**d**), and whole plant (**e**), and P accumulation rates in the whole plant (**f**) of peanut cultivars throughout the season. ** and * are: significant at *p* ≤ 0.01 and *p* ≤ 0.05, respectively, by the F test. Vertical bars indicate the least significant difference values according to the LSD test at *p* ≤ 0.05.

**Figure 4:**
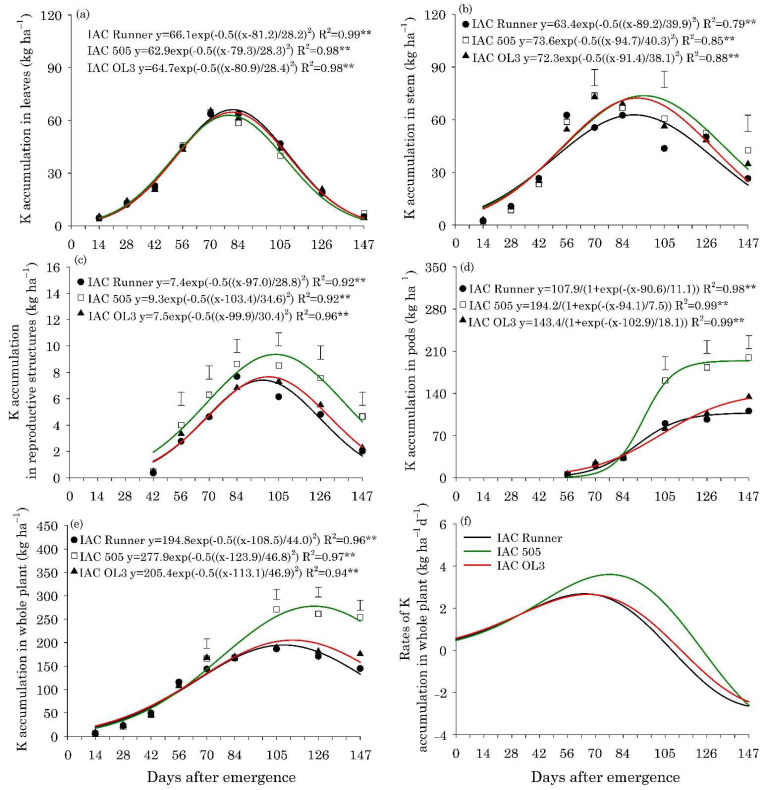
Potassium (K) accumulation in leaves (**a**), stems (**b**), reproductive structures (**c**), pods (**d**), and whole plant (**e**), and K accumulation rates in the whole plant (**f**) of peanut cultivars throughout the season. ** is: significant at *p* ≤ 0.01, by the F test. Vertical bars indicate the least significant difference values according to the LSD test at *p* ≤ 0.05.

**Figure 5:**
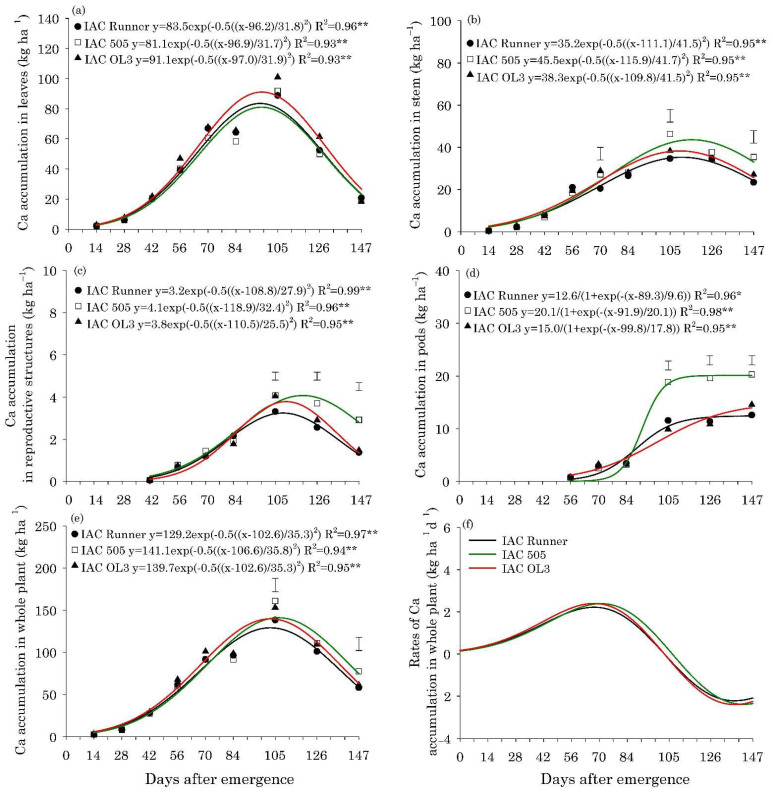
Calcium (Ca) accumulation in leaves (**a**), stems (**b**), reproductive structures (**c**), pods (**d**), and whole plant (**e**), and Ca accumulation rates in the whole plant (**f**) of peanut cultivars throughout the season. ** and * are: significant at *p* ≤ 0.01 and *p* ≤ 0.05, respectively, by the F test. Vertical bars indicate the least significant difference values according to the LSD test at *p* ≤ 0.05.

**Figure 6:**
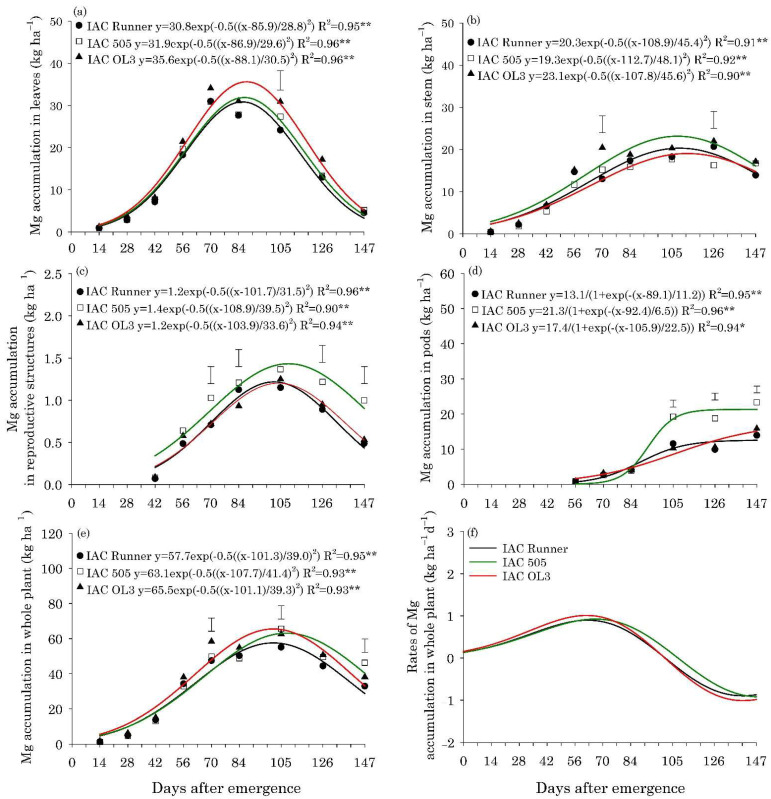
Magnesium (Mg) accumulation in leaves (**a**), stem (**b**), reproductive structures (**c**), pods (**d**), and whole plant (**e**), and Mg accumulation rates in the whole plant (**f**) of peanut cultivars throughout the season. ** and * are: significant at *p* ≤ 0.01 and *p* ≤ 0.05, respectively, by the F test. Vertical bars indicate the least significant difference values according to the LSD test at *p* ≤ 0.05.

**Figure 7:**
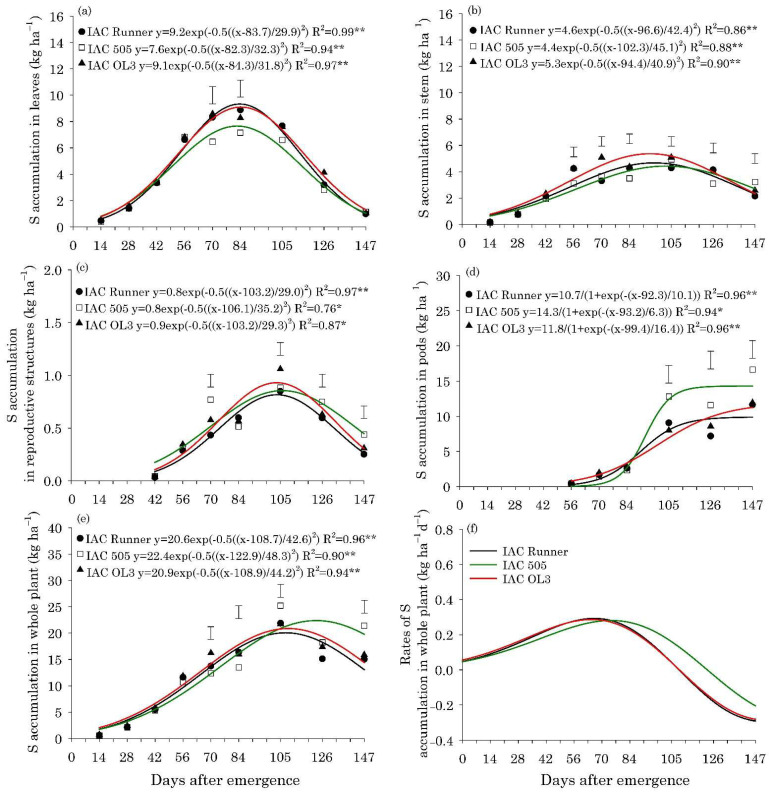
Sulfur (S) accumulation in leaves (**a**), stems (**b**), reproductive structures (**c**), pods (**d**), and whole plant (**e**) and S accumulation rates in the whole plant (**f**) of peanut cultivars throughout the season. ** and * are: significant at *p* ≤ 0.01 and *p* ≤ 0.05, respectively, by the F test. Vertical bars indicate the least significant difference values according to the LSD test at *p* ≤ 0.05.

The maximum accumulation of N, P, K, Mg, and S in the leaves of the three peanut cultivars occurred between 80 and 85 DAE, with approximately 132, 8.0, 65, 33, and 8.6 kg ha^−1^, respectively (Figure 2a, Figure 3a, Figure 4a, Figure 6a and Figure 7a).The maximum accumulation of Ca in the leaves (85 kg ha^−1^) occurred 96 DAE, with this nutrient being the second most accumulated in the peanut leaves (Figure 5a). In the stems, the peak of N, P, K and S accumulation occurred between 90 and 98 DAE, with 57, 4.6, 70, and 4.8 kg ha^−1^, respectively (Figure 2b, Figure 3b, Figure 4b and Figure 7b). For Ca and Mg, the maximum accumulation in the stems occurred at 110 DAE, with 40 and 21 kg ha^−1^, respectively (Figure 5b and Figure 6b).

After 105 DAE, the accumulation of N, P, K, and Ca in the stem of the cultivar IAC 505 stood out, mainly in comparison with IAC Runner (Figure 2b, Figure 3b, Figure 4b and Figure 5b). The greatest N, P, K, Mg, and S accumulation in the reproductive structures occurred between 93 and 105 DAE (Figure 2c, Figure 3c, Figure 4c, Figure 6c and Figure 7c), while for Ca, the highest accumulation was at 113 DAE (Figure 5c). Cultivar IAC 505 tended to have superior accumulation of nutrients in the reproductive structures throughout the season of. Starting at 105 DAE, the greatest accumulation of all nutrients in the pods (Figure 2d, Figure 3d, Figure 4d, Figure 5d, Figure 6d and Figure 7d) and whole plant (Figure 2e, Figure 3e, Figure 4e, Figure 5e, Figure 6e and Figure 7e) of the cultivar IAC 505 was evident.

The accumulation rates of N (8.0 kg ha^−1^ d^−1^), P (1.2 kg ha^−1^ d^−1^), and K (3.5 kg ha^−1^ d^−1^) in the whole plant for IAC 505 were approximately 60%, 33%, and 40% greater than the other cultivars, respectively (Figure 2f, Figure 3f and Figure 4f). The cultivar IAC 505 had the maximum accumulation rate of N (84 DAE), P (63 DAE), and K (77 DAE) later than the cultivars IAC Runner and IAC OL3, with an average of 77 DAE for N, 50 DAE for P and 70 DAE for K. The maximum accumulation rate of Ca occurred at 70 DAE for all cultivars, with 2.0 kg ha^−1^ d^−1^ (Figure 5f) and S with 0.3 kg ha^−1^ d^−1^ (Figure 6f), while for Mg it was at 63 DAE with 1.0 kg ha^−1^ d^−1^ (Figure 7f).

The cultivar IAC 505 showed the greatest productivity of pods and kernels, followed by IAC OL3 and IAC Runner, respectively (Table 2). On average, IAC 505 produced 41% more pods and kernels than IAC OL3, and 69% more pods and kernels than IAC Runner. For each megagram of pods and kernels produced, IAC Runner absorbed more N, K, Ca, Mg, and S than IAC 505 (Table 2). The cultivar IAC Runner absorbed 68.7, 35.5, 23.5, 10.6, and 3.7 kg of N, K, Ca, Mg, and S, respectively, for each megagram of pods produced, and 84.5, 43.7, 28.9, 13.0, and 4.6 kg of N, K, Ca, Mg, and S, respectively, for each megagram of kernels produced.

These values represent an increase of approximately 22%, 18%, 54%, 56%, and 54% more in the absorption of N, K, Ca, Mg, and S, respectively, compared with IAC 505. The cultivar IAC OL3 showed lower contents of P and S in pods and peanut kernels, while IAC 505 showed higher contents of these nutrients. Phosphorus contents in pods and kernels were lower in IAC OL3 than that in the other cultivars, whereas S contents in the same plant structures were lower in IAC 505 and OL3 (Table 2).

The greatest removal of macronutrients by pods (N > K > P > Mg > Ca > S) and by kernels (N > K > P > Mg > S > Ca) occurred by the cultivar IAC 505 (Table 2). For the pods, removal of N (405.9 kg ha^−1^) K (199,7 kg ha^−1^), P (29.5 kg ha^−1^), Mg (23.3 kg ha^−1^), Ca (20.3 kg ha^−1^) and S (16.6 kg ha^−1^) by IAC 505 was greater than the average of the other two cultivars (N: 274.5 kg ha^−1^, K:122.5 kg ha^−1^, P: 18.5 kg ha^−1^, Mg: 14.9 kg ha^−1^, Ca:13.6 kg ha^−1^, S: 11.7 kg ha^−1^).

For each ton of pods or kernels produced, the removal of S by the cultivar IAC Runner was approximately 19% higher than that by the other cultivars (Table 2). From all the N absorbed by peanuts, IAC OL3 showed the highest relative removal by pods (88.8%) and kernels (84.3%) (Table 2).

The relative removal of K, Ca, Mg and S by the pods (71.8%, 14.4%, 37.0%, and 74.3%, respectively) and kernels (65.7%, 9.3%, 32.6%, 65.9%, respectively) was higher in IAC 505 compared with the other cultivars.

Due to the higher accumulation of plant DM, higher pod and kernel yield for the cultivar IAC 505, this cultivar resulted in superior removal of macronutrients by area. On average, IAC 505 removed about 48% N, 64% K, 59% P, 59% Mg, 58% Ca and 44% S more than those removed from the other cultivars. Except for N and P, the relative removal of nutrients was greater in the cultivar IAC 505. The average relative removal of nutrients by the pods, followed the order: P (83%) > N (77%) > K (65%) > S (63%) > Mg (28%) > Ca (12%).

## 3. Discussion

The differences among peanut cultivars for leaf contents of K, Ca and Mg can be explained by the genotypic variation of these materials. In addition, the highest leaf K contents in the cultivars IAC Runner and IAC 505 reflected in the lowest Mg contents in these same cultivars, as there is an antagonism between K and Mg in plants [18]. However, all cultivars presented leaf contents of macronutrients considered adequate for peanuts (N: 30 to 45 g kg^−1^; P: 2.0 to 5.0 g kg^−1^; K: 17 to 30 g kg^−1^; Ca: 12 to 20 g kg^−1^; Mg: 3.0 to 8.0 g kg^−1^ and S: 2.0 to 3.5 g kg^−1^) [12].

Dry matter accumulation in the pods of the cultivar IAC 505 was greater between 105 and 147 DAE, also resulting in a greater DM accumulation in the plants for this same period. The most intense period in the DM accumulation in peanut plants occurs at the beginning of fruiting [8]. According to the authors, an appropriate amount of nutrients should be available in the soil for the plants at the fruiting period. For IAC 505, maximum accumulation of plant DM (30% higher) and maximum accumulation rate (26% higher) occurred approximately ten days later than those for the other cultivars, contributing to its greater productive potential. However, a longer growth duration can represent more time for dry matter production and accumulation [8].

The cultivar IAC 505 accumulated greater macronutrient contents in the pods as well as N, P, and K in the plants between 105 and 147 DAE. The greater accumulation of these nutrients was likely due to the greater DM accumulation both in the pods and the whole plant between 105 and 147 DAE [19]. After the onset of flowering, the peanut crop has a high nutritional demand, and environmental conditions, nutrient supply and management should be adequate to enhance productivity [20]. Nutrient burnout promoted by to insufficient inputs may further limit crop yield. The amount and balance of nutrients in the plants is important to the soil nutrient management strategy [21].

Older peanut cultivars, for example, IAC Caiapó, have the highest growth rate and nutrient accumulation between 100 and 110 DAE [22]. On the other hand, newer cultivars (e.g., IAC Runner, IAC 505 and IAC OL3) have the peak of growth rate and nutrient accumulation earlier, between 70 and 84 DAE. This information is relevant, because quantify of nutrient uptake and of accumulation during the phases of plant development allows for identifying the times at which elements are required most and their distribution in the different structures of the plant, favoring adequate fertilization management [11,14].

The greater productive potential of the cultivar IAC 505 was confirmed. This cultivar was more productive than IAC Runner and IAC OL3 in approximately 3700 and 2700 kg ha^−1^ of pods, respectively. Carrega et al. [23] reported that IAC 505 stood out among 12 genotypes evaluated under well-watered conditions by its highest photosynthetic rate and greater pod production.

For each ton of peanut pods produced, the macronutrient absorption follows the decreasing order: N > K > Ca > Mg > P > S [19], supported by this study. Work reported by [19] indicated that the Penápolis, considered an old cultivar, absorbed 68.2, 19.7, 9.7, 6.7, 3.8, and 2.4 kg of N, K, Ca, Mg, P, and S, respectively, for each ton of pods produced. This information demonstrates that for older cultivars, such as IAC Runner, N absorption remains high, while newer cultivars (e.g., IAC 505 and IAC OL3) are less demanding in N. It is important to highlight that peanuts are legumes; therefore, they have the ability to acquire nitrogen through the process of biological nitrogen fixation (BNF), through symbiosis with bacteria, especially of the genus *Rhizobium* and *Bradyrhizobium*, naturally present in the soil [24].

In contrast, the cultivars used in this study absorbed an average of 65 and 107% more K and Ca than the old cultivars, respectively. Potassium is the second most absorbed nutrient by peanut plants [20], while Ca is the third most absorbed [25]. The greater need for K and Ca by newer modern peanut cultivars suggests that they are more responsive to soil acidity correction by liming, thus providing Ca and improving the availability of Mg for the crop. The large-seeded runner-type cultivars require a higher soil Ca sufficiency level than the smaller-seeded types [26]. The reduction in soil acidity by application of lime favors the increase in the uptake of P and Ca by the plant, resulting in greater pod production of peanuts [27]. It was found that the level of accumulation of K in the plants was much higher in relation to the applied dose of the nutrient (K_2_O - 40 kg ha^−1^). The main explanation is related to the K content of the soil (7.7 mmol_c_ dm^−3^), considered very high [28], being, therefore, sufficient to meet the demand of the crop. Another aspect is that peanuts manage to use the residual fertilizer from the previous crop, being excellent for crop rotations [28]. However, it is noteworthy that it is necessary to replace at minimum the nutrients exported with the grains, through the management of fertilization, for the maintenance of soil fertility [29] and sustainability of agricultural systems [30]. According to [9], the peanut cultivation system has been improved in the past few years, especially due to cultivars being more responsive to fertilizers and changes in management with crop rotation with sugarcane, favoring the potential of the crop production.

## 4. Materials and Methods

### 4.1. Site, Climate, and Soil

A field experiment was conducted in Botucatu, São Paulo, in southeast Brazil (48°23′ W, 22°51′ S, WGS84; 765 m a.s.l.) during the 2014/2015 growing seasons in a Red Latosol [31] or thermic Dystroferric Red Oxisol [32] that has a clayey texture. The climate of the region is Cwa, which is tropical with a dry winter and hot and rainy summer, according to the Köppen classification [33]. Meteorological data during the growing season is shown in Table 3.

Prior to the experiment implementation, a soil sample consisting of 15 subsamples was taken from the 0–0.20 m depth to determine soil chemical properties, according to [34]. The results obtained were: pH [CaCl_2_ 0.01 mol L^−1^ suspension (1:2.5 soil/solution)] = 5.9, organic matter (colorimetric method using a sodium dichromate solution) = 30 g dm^−3^, P (ion exchange resin) = 60 mg dm^−3^, K^+^ = 7.7 mmol_c_ dm^−3^, Ca^2+^ = 59 mmol_c_ dm^−3^, Mg^2+^ = 38 mmol_c_ dm^−3^, H+Al = 26 mmol_c_ dm^−3^, the ions were extracted using an ion exchange resin, CEC = 131 mmol_c_ dm^−3^, base saturation (calculated from exchangeable base content, and total acidity was measured at pH 7.0 (H + Al)) = 80 %, S-SO_4_ (extraction by calcium phosphate 0.01 mol L^−1^ in a 1:2.5 soil:solution ratio and later determined by the turbidimetric method, using BaSO_4_) = 24 mg dm^−3^; B (was carried out using hot water and determined by atomic absorption spectrophotometry) = 0.36 mg dm^−3^; Cu = 12.6 mg dm^−3^; Fe = 16.0 mg dm^−3^; Mn = 34.4 mg dm^−3^; and Zn = 2.6 mg dm^−3^; these micronutrients were evaluated using a 0.005 mol L^−1^ solution of DTPA at pH 7.3, followed by determination by atomic absorption spectrophotometry (Shimadzu model AA-6300; Kyoto, Japan).

### 4.2. Experimental Design and Treatments

The experiment was arranged in a randomized complete block design with split-plots and nine replications. Plots consisted of three peanut cultivars (IAC Runner 886, IAC 505, and IAC OL3), and subplots consisted of the plant samplings (assessments), which occurred at 14, 28, 42, 56, 70, 84, 105, 126, and 147 days after emergence (DAE). Each plot had six 8 m long rows of peanut, spaced at 0.90 m, and each sampling was performed by collecting 0.90 m row length, considering only the four central rows of the plot.

### 4.3. Cultivar Characteristics

The cultivar IAC Runner 886 has a low growth habit, with a cycle of 125 to 130 days, kernels with pink tegument, and yield of 18 to 20 kg kernels per 25 kg bag of shelled peanuts. It has a predominance of commercial kernels classified in sieves 24 and 26, an average of two kernels per pod, oil content in the kernels around 46–48%, and productive potential of 6500 kg ha^−1^ [35].

The cultivar IAC 505 has a low growth habit, is moderately resistant to leaf diseases, and has a cycle of 130 to 140 days. The pods have two medium-sized kernels on average, and kernels have a light brown testa. The productive potential of this cultivar is 6500 kg ha^−1^ and it stands out for the reason that its kernel oil content is greater than that in other cultivars (50%), making it well regarded for both food markets [36,37].

The cultivar IAC OL 3 is indicated for technified systems, which has low vines and an average cycle of 130 days, being indicated for production in areas of renovation of sugarcane fields. The pods have two medium- to large-sized kernels on average as well as pink-colored testa, and the productive potential of IAC OL 3 is approximately 7000 kg ha^−1^. The kernels also have high oil content, approximately 46–47% [36,37].

### 4.4. Crop Management

For the experiment implementation, soil in the experimental area was conventionally prepared, with two heavy gradations followed by two light gradations for leveling the soil. Peanut cultivars were sowed mechanically on December 8th 2014 using 14 seeds m^−1^. In-furrow fertilizer was applied at 20 kg ha^−1^ N, 70 kg ha^−1^ P_2_O_5_, and 40 kg ha^−1^ K_2_O, using NPK fertilizer 08-28-16. No S or micronutrients were applied. Seedling emergence occurred 10 days after sowing (DAS), when more than 50% of the plants in each plot emerged from the soil surface.

At 23 DAE, the herbicide imazepique was applied at a rate of 145 g a.i. ha^−1^, and at 37 and 49 DAE the herbicide haloxyfop-methyl ester was applied at a rate of 60 g a.i. ha^−1^ for weed control. The peanut crop was cultivated under dryland conditions. Throughout the season, agricultural practices were performed as needed and followed recommendations given in [36].

### 4.5. Plant Measurements and Analysis

For evaluation of plant nutritional status, 10 plants were sampled per subplot (apical cluster of the main branch) at the full-bloom stage according to protocol described by [12]. Plant tissue was dried in an oven at 65 °C until constant weight and then ground for macronutrient analyses. The contents of N, P, K, Ca, Mg, and S were determined using methods described by [38]. Nitrogen was determined by the Kjeldahl method (Tecnal model TE-036/1; Piracicaba, São Paulo, Brazil). For the determination of other nutrients, milled plant material was mineralized with a nitric-perchloric solution. From this solution, K, Ca, and Mg contents were determined using an atomic absorption spectrophotometer. P and S were measured by colorimeter methods using a spectrophotometer (Shimadzu model UV-2700; Kyoto, Japan) [38].

For assessment of dry matter (DM) and nutrient accumulation by the peanut cultivars, the sampled plants were separated into leaves, stems, reproductive structures (flower plus gynophore), and pods, and were washed and dried until at constant weight in a forced-air oven at 65 °C. Based on the DM data and plant density, the amounts of DM accumulated in each plant tissue were calculated, and DM accumulated in the whole plant was obtained by summing the amounts of DM accumulated in each plant tissue. The samples were ground in a Willey mill (Marconi model 340; Piracicaba, São Paulo, Brazil). and the contents of N, P, K, Ca, Mg, and S were determined [38]. Based on the nutrient contents and amounts of DM accumulated, the amounts of macronutrients accumulated in each plant tissue were calculated. The amounts of macronutrients accumulated in each plant tissues were summed to obtain the amounts of macronutrients accumulated in the whole plant. Accumulation rates of DM and macronutrients in the whole plant were obtained by the first derivative of the adjustment equations. The amounts of macronutrients taken up per ton of pods and kernels produced were obtained by dividing the maximum amounts of macronutrients accumulated in the whole plant by pod or kernel yield.

### 4.6. Pod and Kernel Yield and Nutrient Removal

Harvest was performed manually on May 13th, 2015. Pod yield (moisture content of 90 g kg^−1^) was determined by manually harvesting the plants from the central row of the subplot. The kernels were then removed from the hulls and weighed to determine kernel yield (moisture content of 90 g kg^−1^). Pod and kernel yield (kg ha^−1^) of peanut cultivars were obtained considering the weight of pods and kernels per plant sampled and the final plant population. A sample of pods and kernels from each plot was dried in a forced air (Fanem model 330; Guarulhos, São Paulo, Brazil) oven at 65 °C for 72 h, and these samples were ground in a Willey mill. The macronutrient contents in the pod and kernel were determined according to [38] and nutrient removal by pods and kernels was derived from the pod and kernel yield data and the nutrient content in pods and kernels. The amounts of macronutrients removed per ton of pods and kernels produced was obtained by dividing the maximum amount of macronutrients removed by pods or kernels by pod or kernel yield. The relative removal was obtained by dividing the maximum amounts of accumulated macronutrients in the whole plant by the amounts removed by the pods or kernels, multiplied by 100.

### 4.7. Statistical Analyses

Data were subjected to ANOVA. The mean values for the cultivars at each sampling time were separated by the LSD test at 0.05 probability. The effects of the plant samplings on the DM and nutrient accumulation variables were assessed by regression analysis using SigmaPlot 10.0 software.

## 5. Conclusions

Our results revealed that modern peanut cultivars presented highest growth rate and nutrient accumulation around 30 to 40 days earlier than older cultivars (reported in the literature), in addition to increasing the requirement for K and Ca. The cultivar IAC 505 presented the highest dry matter production potential in all plant tissues, including pods and kernels, as well as the highest uptake of macronutrients, while IAC OL3 and IAC Runner 886 were similar to each other. Our results reinforce that modern peanut cultivars stand out compared with the older ones. In addition, our work brought new possibilities regarding the greater responsiveness of modern peanut cultivars to soil acidity correction with Ca-based amendments, as well as the most appropriate time for fertilization practices, indicating new perspectives for updating and improving nutritional management recommendations for high peanut yields. Further research with modern peanut cultivars should be encouraged, especially with the application of Ca-based additives and fertilizer rates, as well as application times, which may generate adequate fertilization recommendations for new cultivars.

## Figures and Tables

**Table 1:** Content of N, P, K, Ca, Mg, and S in leaves of peanut cultivars at the full-bloom stage.

Cultivars	N	P	K	Ca	Mg	S
	^______________________________________^g kg^−1______________________________________^					
IAC Runner	36 a	2.5 a	20 a	22 a	6.2 c	2.5 a
IAC 505	38 a	2.5 a	20 a	20 ab	6.8 b	2.3 a
IAC OL3	36 a	2.4 a	17 b	19 b	7.4 a	2.6 a
CV%	5.7	9.1	6.2	5.4	4.1	6.7

Values followed by the same letter in the column are not significantly different at *p* ≤ 0.05 according to the LSD test.

**Table 2:** Pod and kernel yield, macronutrient taken up by megagram of pods and kernels produced, macronutrient content in pods and kernels, macronutrient removal by area and by megagram of pods and kernels produced, and relative macronutrient removal by pods and kernels of peanut cultivars.

Cultivar	Yield (kg ha^−1^)		N	P	K	Ca	Mg	S
	Pods	Kernels						
			Macronutrient taken up by Mg of pods produced (kg Mg^−1^) ^(1)^					
IAC Runner	5489 c	4462 c	68.7 a	4.0 a	35.5 a	23.5 a	10.6 a	3.7 a
IAC 505	9230 a	7603 a	56.2 b	3.9 a	30.1 b	15.3 b	6.8 b	2.4 b
IAC OL3	6492 b	5445 b	53.1 b	3.4 b	31.7 a	21.4 ab	10.0 a	3.2 ab
			Macronutrient taken up by Mg of kernel produced (kg Mg^−1^) ^(1)^					
IAC Runner	-	-	84.5 a	4.9 a	43.7 a	28.9 a	13.0 a	4.6 a
IAC 505	-	-	68.3 b	4.8 a	36.6 b	18.5 b	8.3 b	2.9 c
IAC OL3	-	-	63.4 b	4.0 b	37.8 a	25.5 ab	11.9 a	3.8 b
			Macronutrient content in pods (g kg^−1^)					
IAC Runner	-	-	44.4 a	3.4 a	20.1 a	2.3 a	2.5 a	2.1 a
IAC 505	-	-	44.0 a	3.2 a	21.7 a	2.2 a	2.5 a	1.8 b
IAC OL3	-	-	47.2 a	2.9 b	20.7 a	2.2 a	2.4 a	1.8 b
			Macronutrient content in kernels (g kg^−1^)					
IAC Runner	-	-	51.8 a	4.0 a	22.4 a	1.7 a	2.7 a	2.3 a
IAC 505	-	-	51.0 a	3.8 a	24.0 a	1.7 a	2.7 a	1.9 b
IAC OL3	-	-	53.4 a	3.3 b	22.4 a	1.5 b	2.5 a	1.9 b
			Macronutrient removal with pods by area (kg ha^−1^)					
IAC Runner	-	-	242.9 c	18.4 b	110.8 b	12.6 b	13.9 b	11.6 b
IAC 505	-	-	405.9 a	29.5 a	199.7 a	20.3 a	23.3 a	16.6 a
IAC OL3	-	-	306.2 b	18.7 b	134.2 b	14.6 b	15.9 b	11.9 b
			Macronutrient removal with kernels by area (kg ha^−1^)					
IAC Runner	-	-	230.4 c	17.9 b	100.8 b	7.4 b	12.0 b	10.1 b
IAC 505	-	-	387.1 a	28.6 a	182.6 a	13.1 a	20.5 a	14.8 a
IAC OL3	-	-	291.0 b	18.0 b	121.9 b	8.4 b	13.4 b	10.2 b
			Macronutrient removal by Mg of pods produced (kg Mg^−1^)					
IAC Runner	-	-	44.4 a	3.4 a	20.1 a	2.3 a	2.5 a	2.1 a
IAC 505	-	-	44.0 a	3.2 a	21.7 a	2.2 a	2.5 a	1.8 b
IAC OL3	-	-	47.2 a	2.9 a	20.7 a	2.2 a	2.4 a	1.8 b
			Macronutrient removal by Mg of kernels produced (kg Mg^−1^)					
IAC Runner	-	-	51.8 a	4.0 a	22.4 a	1.7 a	2.7 a	2.3 a
IAC 505	-	-	51.0 a	3.8 a	24.0 a	1.7 a	2.7 a	1.9 b
IAC OL3	-	-	53.4 a	3.3 a	22.4 a	1.5 b	2.5 a	1.9 b
			Relative macronutrient removal by pods (%) ^(2)^					
IAC Runner	-	-	64.4	83.8	56.8	9.8	24.0	56.7
IAC 505	-	-	78.2	81.4	71.8	14.4	37.0	74.3
IAC OL3	-	-	88.8	85.1	65.2	10.5	24.4	57.9
			Relative macronutrient removal by kernels (%) ^(2)^					
IAC Runner	-	-	61.1	81.3	51.7	5.8	20.7	49.4
IAC 505	-	-	74.6	79.1	65.7	9.3	32.6	65.9
IAC OL3	-	-	84.3	82.0	59.2	6.0	20.6	49.7

Values followed by the same letter in the column within each parameter are not significantly different at *p* ≤ 0.05 according to the LSD test. ^(1)^ Data based on pod and kernel yield and on the values of maximum macronutrient accumulated in the whole plant, shown in Figure 2e, Figure 3e, Figure 4e, Figure 5e, Figure 6e and Figure 7e. ^(2)^ Proportional macronutrient removal by pods or kernels in relation to the maximum amounts of macronutrient taken up by peanut cultivars, shown in Figure 2e, Figure 3e, Figure 4e, Figure 5e, Figure 6e and Figure 7e.

**Table 3:** Rainfall, maximum and minimum temperatures received at Botucatu, São Paulo, Brazil, during the study period and averages.

Climate Characteristics	Month					
	December	January	February	March	April	May
	2014/2015					
Monthy rain (mm)	265	256	252	265	46	99
Mean max. Temp (°C)	28.6	31.7	28.4	27.1	27.0	23.4
Mean min. Temp (°C)	15.5	19.1	18.1	17.2	16.1	13.4

## Data Availability

The data presented in this study are available on request from the corresponding author. The data are not publicly available due to they belong to a network of experiments.
